# Nanolitre-scale crystallization using acoustic liquid-transfer technology

**DOI:** 10.1107/S0907444912016617

**Published:** 2012-07-17

**Authors:** Armando G. Villaseñor, April Wong, Ada Shao, Ankur Garg, Timothy J. Donohue, Andreas Kuglstatter, Seth F. Harris

**Affiliations:** a Department of Discovery Technologies, Roche Palo Alto LLC, 3431 Hillview Avenue, Palo Alto, CA 94304, USA; b EDC Biosystems, 49090 Milmont Drive, Fremont, CA 94538, USA; c Department of Discovery Technologies, F. Hoffmann-La Roche, 4070 Basel, Switzerland; d Department of Structural Biology, Genentech, 1 DNA Way, South San Francisco, CA 94080, USA

**Keywords:** acoustic liquid transfer, nanolitre-scale crystallization

## Abstract

Focused acoustic energy allows accurate and precise liquid transfer on scales from picolitre to microlitre volumes. This technology was applied in protein crystallization, successfully transferring a diverse set of proteins as well as hundreds of precipitant solutions from custom and commercial crystallization screens and achieving crystallization in drop volumes as small as 20 nl. Only higher concentrations (>50%) of 2-­methyl-2,4-pentanediol (MPD) appeared to be systematically problematic in delivery. The acoustic technology was implemented in a workflow, successfully reproducing active crystallization systems and leading to the discovery of crystallization conditions for previously uncharacterized proteins. The technology offers compelling advantages in low-nanolitre crystallization trials by providing significant reagent savings and presenting seamless scalability for those crystals that require larger volume optimization experiments using the same vapor-diffusion format.

## Abstract 2

Acoustic droplet ejection achieves precise, tipless, non-invasive transfer of diverse aqueous solutions, enabling nanolitre-scale crystallization trials. The rapid and scalable technique demonstrated successful crystal growth with diverse targets in drop volumes as small as 20 nl.

## 1. Introduction

Crystallization experiments, as with many scientific fields, have followed a trend towards increased automation and miniaturization to improve efficiency and accelerate discovery (Stevens, 2000 ▶). Tip-based liquid dispensing, the natural extension of the most common manual tools, has been a robust method for setting up crystallization trials well into the sub­microlitre range, with typical volumes of 100–200 nl (*e.g.* Mosquito by TTP LabTech, Royston, England, Phoenix by Art Robbins, Sunnyvale, Califonia, USA and Crystal Creator by Cybio AG, Jena, Germany, among others). Yet smaller volumes are enabled by inkjet-type solenoid dispensers (Rose, 1999 ▶), which aspirate liquid into a tubing line and dispense from a tip without making contact with the destination surface. Such non-contact dispensers still contact the sample at the dispensing head and as such are still prone to surface-contact artifacts, clogs and possible cross-contamination from viscous solutions (Walter *et al.*, 2003 ▶). Microfluidic methods also achieve small-volume experiments (∼10 nl), but are constrained by the physical parameters of the fluidics channels and the resulting impact on crystal harvesting and challenges in translation from free-interface diffusion, for example, to more conventional methods (Fluidigm, South San Francisco, California, USA and Plugmaker, Emerald Bio­Systems, Bainbridge Island, Washington, USA). Acoustic liquid transfer, as implemented in the ATS-100 (EDC Biosystems, Fremont, California, USA) and the Echo series instruments (Labcyte, Sunnyvale, California, USA), uses focused sound energy to eject single-digit nanolitre droplets directly from a source-liquid meniscus up onto inverted target destinations with no physical contact with the sample. In prior review, acoustic dispensing showed unmatched precision (<2% CV) at sub-100 nl volumes compared with several of the technologies described above (Comley, 2004 ▶). Furthermore, the lack of fluidic lines and tips greatly reduces consumables, maintenance, washing steps, clogs and cross-contamination.

To achieve acoustic liquid transfer, a small transducer bathed in a coupling fluid (*e.g.* water) to effect efficient energy transfer is positioned under a micro plate (‘source plate’). Acoustic reflections can determine the liquid level in the source wells and thereby the appropriate focal distance to position the transducer. The focused energy leads to deformations at the liquid–air interface and the ejection of nanolitre-scale droplets on a millisecond time scale. These droplets travel upwards to contact and accumulate on an inverted target plate, building up to the prescribed volume. The technology has rapidly expanded in the realm of small-molecule compound library management and high-throughput screening groups, where the compounds of interest are typically in a well characterized organic solvent such as dimethyl sulfoxide (DMSO; Ellson *et al.*, 2005 ▶; Olechno *et al.*, 2006 ▶). Further research has also demonstrated its utility in microarray setups (Wong & Diamond, 2009 ▶) and MALDI imaging mass spectroscopy (Aerni *et al.*, 2006 ▶).

Motivated by the advantages described above and a potential saving of an order of magnitude in protein consumption relative to other vapor-diffusion methods, we explored the application of this technology in protein crystallography. A primary concern was whether the instrument, which was calibrated for performance with DMSO, would work acceptably with the diverse range of aqueous solutions involved in typical crystallization screening. We anticipated that the well-by-well energy tuning available on the ATS-100 instrument would permit us to mitigate the impact of such distinct physicochemical properties of the crystallization cocktails (*e.g.* viscosity and surface tension).

## 2. Experimental procedures

### 2.1. Hardware and consumables

Standard tip-based liquid dispensing (Cybiwell liquid handler from Cybio AG, Jena, Germany) was employed in advance to fill the large reservoirs in crystallization plates (Corning 3785 96-well sitting-drop vapor-diffusion plate; Corning Life Sciences, Lowell, Massachusetts, USA) with 25 µl per well and also to fill the source plates (384 IQ-LV, 200 µm; Aurora Biotechnologies, Carlsbad, California, USA) with custom and commercial screens with 15 µl per well. All acoustic liquid transfers were performed on the EDC Biosystems ATS-100. The protein sample to be transferred was pipetted into a single well (15 µl) in the source plate just before the start of the acoustic run. A gripper inverts the crystallization plate (target) over the source plate during transfer, with each plate free to translate relative to the other such that any source well can be addressed to any of the target wells. We designed our acoustic setups to deliver a first pass transferring the precipitant solutions (typically 96 wells, each delivering 20 nl) followed by a second pass delivering the protein (typically 20 nl) on top of each of the just-dispensed 96 precipitant solutions. The volumetric ratio of precipitant to protein was predefined by the user with standard text-file inputs (*i.e.* ‘ejection maps’) designating volume and destination wells for each liquid. Similarly, ‘energy maps’, used in some of our experiments, specified the amplitude and time base of the acoustic energy pulse for each separate well. In contrast, for the fixed-energy benchmark used in most of the experiments described here the instrument was set to an amplitude of 1.6 and a burst of 350 (manufacturer’s scale) for all wells. All crystallization drops were assembled with the instrument’s volume calibration at 5 nl delivery per energy pulse. The entire setup of one 96-well crystallization plate was achieved in 2.5 min. The completed plate was immediately sealed with CrystalSeal Film (Hampton Research, Aliso Viejo, California, USA) to minimize evaporation. Source-plate and target-plate geometries were entered using well-spacing dimensions from the manufacturers with distal corners as fiducial markers. Additionally, a flat-surface micro-plate lid (Corning 3950) was used as an alternative target when testing individual liquids and their ability to transfer.

### 2.2. Transfer-energy adjustments based on acoustic liquid-level measurements

The instrument uses acoustic reflection to measure liquid levels in each source well. We used these data to establish liquid levels before and after dispensing 200 droplets for each well and thereby the average change in volume per single drop ejection. We tested dispensing in five different screens: Nextal JCSG+, Hampton Research Index HT, Nextal Classics, Emerald BioSystems Wizards I and II, and an in-house medium-weight polyethylene glycol grid [‘PEG MW’, sampling 5, 15, 20 and 25%(*w*/*v*) PEG 3350, PEG 5000 MME and PEG 6000 across the pH range 3.5–9.8 using the buffer systems sodium lactate, sodium acetate, 2-(*N*-morpholino)­ethanesulfonic acid (MES), imidazole, potassium phosphate, 4-(2-hydroxyethyl)-1-piperazineethanesulfonic acid (HEPES), 2-[(2-aminoacetyl)amino]acetic acid (Gly-Gly) and glycine]. Each sample was run six times (two source-plate replicates, each run three times) and the average drop volume was determined. The residual difference from the target value of 5 nl was calculated (*x* in the equations below) and was used to set a revised burst energy from an empirically derived adjustment factor as a proof-of-concept gauge of improved accuracy of delivery. Liquids that lacked a liquid-level reading were excluded. For liquids that delivered more than 5 nl (*i.e.* *x* < 0) the new burst was given by
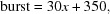
while for liquids that delivered less than 5 nl (*x* > 0) the new burst was given by
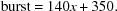
A linear slope extrapolation was used for simplicity, anticipating more sophisticated fits based on knowledge learned. The coefficients of variation were calculated by standard methods,
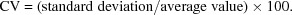



### 2.3. Viscosity measurements

The viscosities and densities of solutions from the JSCG+ and Classics screens were determined using a 30-tube sampler to feed an Anton Paar SP3-V viscosity meter in tandem with a DMA4500 density meter (Anton Paar, Graz, Austria). A total of 10 ml of each solution was loaded into 12 ml glass vials in the auto sampler. Average values were calculated from a total of four individual measurements per solution at 293 K. Each viscosity measurement was based on the free-fall duration of a 1.5 mm diameter gold-plated steel ball in a 1.6 mm diameter capillary 100 mm in length with an inclination of 60°. Free-fall durations were marked by an inducible coil sensor at both ends of the capillary.

### 2.4. Protein reagents

Six proteins with known crystallization conditions were employed to evaluate the reproduction of crystallization in acoustically dispensed drops. These proteins were hepatitis C virus helicase (HCV helicase), human serum albumin (HSA), hepatitis C virus RNA-dependent RNA polymerase (HCV polymerase), human immunodeficiency virus reverse transcriptase (HIV RT), human IL2-inducible T-cell kinase (ITK) and chicken egg-white lysozyme. Visible protein crystals were obtained as follows. For HCV helicase (purification modeled as described in Kim *et al.*, 1998 ▶), we assembled crystallization drops with 15 or 25 nl 15%(*w*/*v*) PEG 6000, 0.2 *M* lithium sulfate, 0.02 *M* magnesium chloride, 0.1 *M* Tris pH 8.0 and 15 or 25 nl 10 mg ml^−1^ protein. The same volumes were used for HSA (catalog No. pro369; ProSpecBio, Rehovot, Israel), where we mixed 27%(*v*/*v*) PEG 550 monomethyl ether, 0.05 *M* magnesium chloride, 0.1 *M* HEPES pH 7.5 and 25 mg ml^−1^ protein in 0.05 *M* potassium phosphate pH 7.2. HCV polymerase drops were assembled with 20 nl 24%(*w*/*v*) PEG 4000, 7.5%(*v*/*v*) glycerol, 0.05 *M* sodium citrate pH 4.6 and 40 nl 10 mg ml^−1^ protein purified as described by Le Pogam *et al.* (2006 ▶). HIV RT drops were formed with 15 nl 1.2 *M* sodium malonate, 5%(*v*/*v*) ethylene glycol, 0.1 *M* potassium phosphate pH 7.2 and 15 nl 10 mg ml^−1^ protein (purified as described previously; Sweeney *et al.*, 2008 ▶) inhibited with 0.25 m*M* nevirapine. ITK drops were set up with 30 nl 25%(*w*/*v*) PEG 3350, 0.2 *M* ammonium acetate, 0.1 *M* HEPES pH 7.5 and 30 nl 10 mg ml^−1^ triple mutant C477S/E614A/E617A (purified as described previously; Kutach *et al.*, 2010 ▶). Lysozyme drops were formed with 15 nl 0.9 *M* sodium chloride, 0.1 *M* sodium acetate pH 5.0 and 15 nl 10 mg ml^−1^ protein (catalog No. L6876; Sigma, Saint Louis, Missouri, USA) dissolved in 0.1 *M* sodium acetate pH 4.6.

The ATS-100 instrument was set at fixed energy for all experiments with these proteins. All vapor-diffusion trials featured large reservoirs filled with only 15 or 25 µl of the corresponding precipitant to prevent liquids from dripping from inverted plates during acoustic dispensing. The experiments were sealed with Clear Seal tape (Hampton Research, Aliso Viejo, California, USA) immediately after setup. The drops were then incubated at 293 K and digitally imaged in a Crystal Farm imager (Nexus Biosystems, Poway, California, USA) on days 0, 3, 5, 10, 15 and 30.

## 3. Results

**Figure 1:**
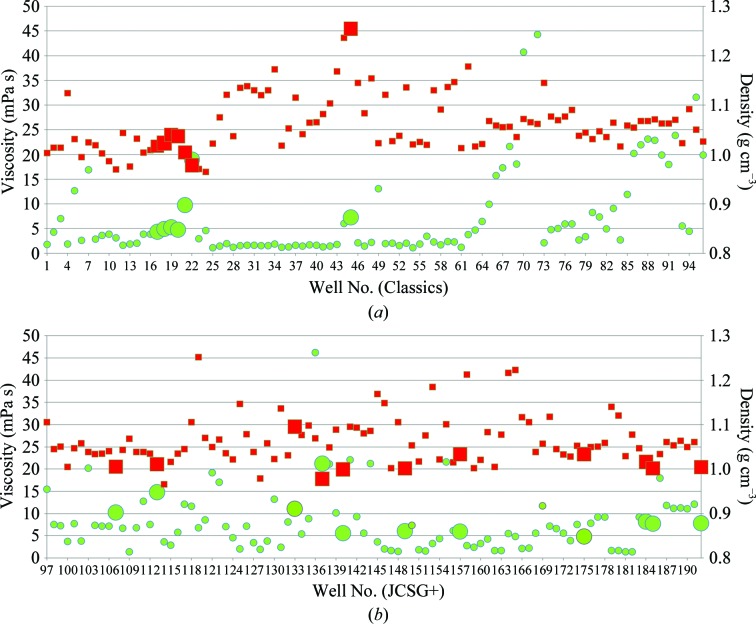
Measurement of viscosity (green circles) and density (red squares) of solutions from the Classics (*a*) and JCSG+ (*b*) screens. The larger symbols represent solutions that failed to dispense and show no clear trend toward either extreme.

Our panel of test screens included several sparse-matrix kits to ensure a good sample of the conditions encountered in crystallization trials. Four 96-­well commercial screens were tested (JCSG+, Index, Classics and Wizards I and II, representing 284 unique conditions; Newman *et al.*, 2010 ▶) as well as a 96-­condition medium-weight PEG grid. Using a fixed energy, 93% (447/480) of the precipitant solutions transferred successfully and the remaining 7% (33 conditions) failed to dispense. We were curious whether the viscosity of the liquid sample was a predictor of poor transfer capabilities. Fig. 1 ▶ shows the viscosities and densities of difficult and well behaved solutions from the Classics and JCSG+ screens, demonstrating that there is no correlation linking viscosity or density alone to inability to dispense. Surface tension is a dominant factor in the mechanism of droplet ejection, but we could not readily characterize this property.

A full description of the contents of the 33 problematic solutions is shown in Table 1 ▶. It was immediately obvious that the majority of the non-transferring conditions (26 out of 33) contained high concentrations of 2-methyl-2,4-pentanediol (MPD). Conditions with MPD at less than 30% concentration could be transferred. Three samples including 30% MPD with observed transfer all contained 0.1 *M* sodium acetate pH 4.6 and a chloride salt, while other buffered 30% MPD solutions failed, as seen in Table 1 ▶. All samples with greater than 30% MPD failed to transfer. Further work has suggested that MPD attenuates acoustic energy and that stronger input enables the delivery of solutions up to ∼50% MPD (J. Bramwell, Labcyte, personal communication). Of the seven non-MPD-containing failed wells, three are represented in other screens, where measurable transfer was observed (marked with asterisks in Table 1 ▶). A few other unusual constituents such as branched polymers such as pentaerythritol propoxylate and polypropylene glycol are present in two non-transferring wells, but as these are less common our data do not inform whether or not their impact is systematic.

**Figure 2:**
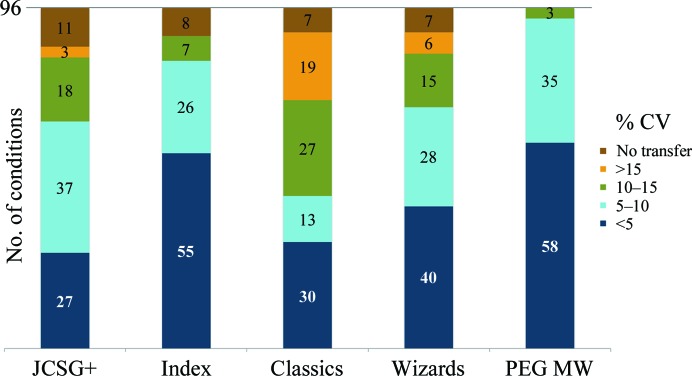
The distribution of coefficients of variation (CVs) for reproducibility in five crystallization screens calculated from six repeat dispenses. Each block of color shows the count of conditions from that screen within the prescribed range. The brown segment tallies wells that did not transfer and hence have no calculated CV.

We wished to characterize the successful liquid transfers for reproducibility and accuracy. The standard calibration method of fluorescein-doped deliveries was impractical for the scale of this work. We used the acoustic reflection measurements of liquid heights (included in the instrument run logs) before and after a series of ejections to determine the volume delivered per droplet compared with the calibrated setting (5 nl). While the effect of the distinct solutions on the speed of sound confounds the absolute accuracy of these measurements, the differential nature of the pre- and post-delivery determination provides a relative difference that ought to be robust. Using six repeat experiments, Fig. 2 ▶ plots the number of conditions in each screen within CV categories binned by 5% increments. Each screen demonstrates that the majority of conditions have good reproducibility (CV < 10%). In addition, the PEG MW screen, with a moderate diversity of components, had superior precision, with 93 of 96 conditions showing CVs of less than 10% (and many of less than 2%). The overlap of conditions in the commercial screens (Newman *et al.*, 2010 ▶) also provided 84 duplicate solutions from different screens with observed transfer. As an additional measure of reproducibility, the mean difference between the measured volume per droplet for these 42 pairs was only 0.22 ± 0.21 nl.

**Figure 3:**
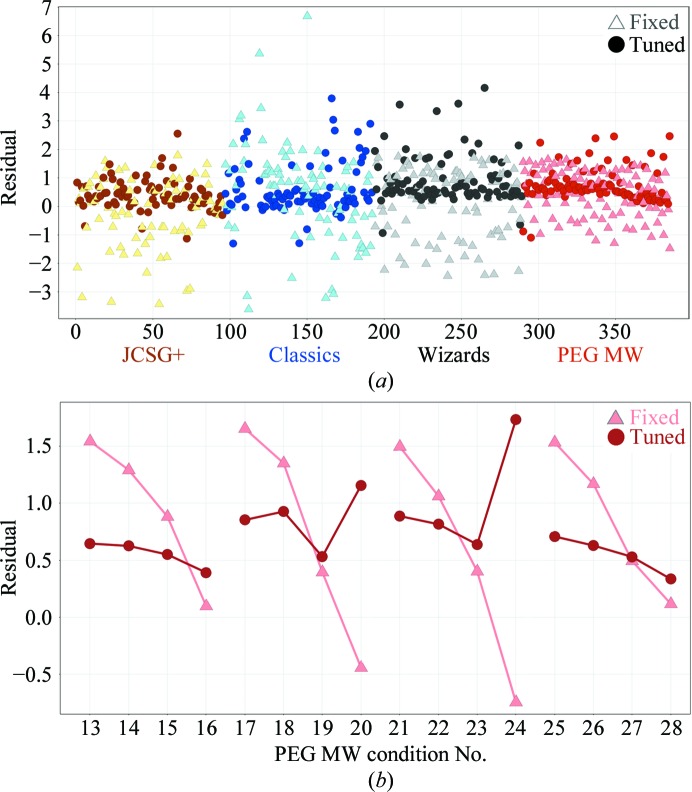
Plots of the residual error in measured volume delivered relative to a 5 nl target for experiments at fixed energy throughout (light triangles) *versus* individually adjusted energies (dark circles). (*a*) Distribution of residuals across four screens (color-coded). (*b*) Enlarged view of conditions 13–28 of the PEG MW grid. Each set of four linked points is a progression of PEG concentration (5, 15, 20, 25%), illustrating the nature of the effect of the diminished measured volumes delivered as PEG increases (triangles). Application of a coarse energy correction (circles) demonstrates better delivery profiles (smaller range in residuals across PEG concentrations) and highlights further improvements to our energy-correction calculations to reduce overcompensation of the lowest measured residuals (*e.g.* points 20 and 24).

We used the measured residual volume differences (*versus* a 5 nl target) to calculate semi-empirical adjusted energy parameters for individual wells to gauge potential improvements *versus* fixed-energy operation. Fig. 3 ▶ plots these residuals from four screens, showing a much tighter distribution when well-by-well energy adjustments were applied. This is quantitated in Table 2 ▶, where the standard deviation over all wells of the absolute volume (*i.e.* not the residual) relative to the mean of the distribution improves significantly from 1.28 to 0.76. Naturally, the more chemically diverse sparse-matrix screens have a higher absolute deviation than the limited chemistry of the PEG MW screen, but the salient metric is in the improvement (smaller standard deviation) in each screen upon adjusted-energy dispenses (Table 2 ▶). Aside from profiling the absolute volume delivered, we gauged accuracy by calculating the root-mean-square deviation of the residuals (relative to the 5 nl target volume). These r.m.s.d.s showed slightly less improvement than the standard deviations of the absolute volumes (residual r.m.s.d. of 1.30 Å at fixed energy to 1.01 Å with ‘tuned’ energies). It is apparent that our coarsely calculated energy adjustments led to a slightly larger average volume delivered (5.23 nl per droplet mean volume at fixed energy, 5.67 nl mean with tuned energies) and this systematic bias, which is purposefully captured in the residual r.m.s.d., partly offsets the gains of the tighter distribution relative to the mean. Adjusting the constant parameter in our admittedly simplistic first-pass energy-adjustment calculations would presumably improve the accuracy. Furthermore, Fig. 3 ▶(*b*) highlights a segment of the PEG MW screen residual plot in which each set of four points represents increasing concentrations of PEG (5, 15, 20 and 25%). Overall, the energy adjustments yield a much flatter range within each group of four data points. Of note, however, is that those highest PEG concentrations (the fourth point of each set) with the most reduced delivery volumes at fixed energy resulted in over-compensation, a phenomenon that accounts for many of the higher scatter points in Fig. 3 ▶(*a*) and contributes to the upward bias in average volumes. The adjustment energies would be likely to improve with an asymptotic curve instead of our linear extrapolation. These studies investigating the relationship between energy parameters and volume delivery of different chemical series helped to inform more sophisticated developments for the acoustic methodologies that are now forthcoming. However, given the complexity of determining and implementing the energy maps in the current format *versus* the practical functionality and emerging successes of the fixed-energy operation, we performed all our subsequent crystallization trials using the simpler fixed-energy setting.

**Figure 4:**
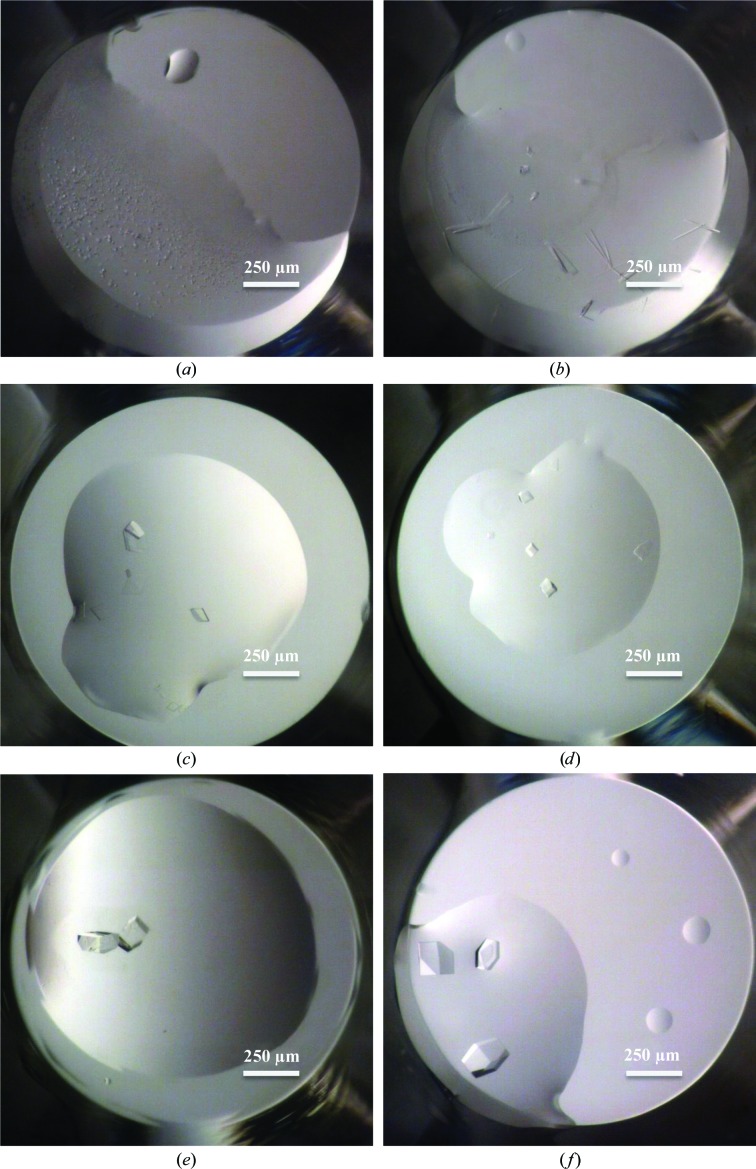
Images collected on day 15 from crystallization drops of various protein samples. The total drop volume is double the protein value given. The volumes are representative and were not optimized as similar crystals appeared at the various volumes explored. (*a*) HCV helicase (50 nl). (*b*) Human serum albumin (50 nl). (*c*) HCV polymerase (30 nl). (*d*) HIV RT (15 nl). (*e*) ITK (30 nl). (*f*) Lysozyme (15 nl).

We continued experiments to verify that diverse protein samples would crystallize using this new application of acoustic technology. In addition to a trivial control (lysozyme), we were able to reproduce the crystallization of HCV helicase, HCV polymerase, HSA, HIV-RT and ITK (five in-­house targets). Crystals of all six proteins were visible by day 5 and most stopped growing by day 15. Fig. 4 ▶ shows typical crystals; various representative volumes were used in these trials but were not a necessary part of optimization.

Finally, we experimented with the delivery of a set of oils to look at alternative ways to reduce evaporation from the small crystallization drops and to consider whether acoustic delivery could enable batch-under-oil methods. We used high energy settings in these tests, typically with amplitude at 2.5–3.5 and burst settings greater than 1000. Silicone oil, DMS (polydimethyl­siloxane terminated with trimethyl­siloxy), FMS [poly(3,3,3-trifluoropropylmethylsiloxane)] and Al’s oil (a 50:50 mixture of silicone and paraffin oil) failed to transfer at these energies. However, we did observe transfer of paraffin oil and mineral oil, but did not quantitate these deliveries. Challenges to using oils in such flying-drop setups remain, as there was noticeable spatter upon aqueous drop and oil surface contact.

## 4. Discussion

Smaller crystallization-drop volumes increase the number of conditions that can be investigated per volume of protein sample. Nanocrystallization thus provides a more efficient use of protein reagents and may afford a thorough crystallization screening of low-expressing targets that were previously intractable with traditional techniques. On the other hand, the altered biophysical environment of such small drops is complex, with a possible detrimental impact on nucleation and thus crystallization. Whether lessened homogeneous nucleation from bulk solution may be offset by increased surface-effect contributions and heterogeneous nucleation is a relevant discussion (Bodenstaff *et al.*, 2002 ▶). In our practice, reproduction of known crystallization systems was relatively straightforward, suggesting that at least at these volumes (20–60 nl) the various biophysical considerations had not shifted too greatly from more traditional scales (*i.e.* 300 nl to single microlitre). Additionally, the delivered volume in an acoustic instrument is provided in a series of millisecond time-scale pulses resulting in ejections of a series of droplets (*e.g.* 5 nl, though even picolitre volumes are technically achievable) that rapidly build to the desired target volume, thus providing continuous access to the whole regime from nanolitre to microlitre volumes. This consistent format mitigates issues that have been encountered in microfluidic free-interface diffusion methods, where reproduction of crystallization in larger, more accessible systems has not always been straightforward.

It was unclear whether the diverse aqueous solutions common to crystallography would transfer acceptably using this technology, which was previously established for well characterized and consistent organic solvents (*e.g.* CV < ∼2% for DMSO solutions). However, the large majority of crystallization solutions (93%) transferred successfully without any energy adjustments. Additionally, reproducibility was robust as 73% of conditions across five screens (349 of 480 conditions) had CV < 10% and 87% of conditions had CV < 15%.

Ultimately, viable crystallization was our desired read-out. We successfully used acoustic droplet ejection to crystallize proteins from our active structure-based drug-discovery projects. This included broad screening to identify a novel crystal form of the protein kinase ITK with characteristics suitable for a structure-based drug-design campaign (Kutach *et al.*, 2010 ▶) and a Fab–protein target complex. In the latter case, a crystal from trials using only 20 nl protein solution per drop was harvested and diffracted sufficiently to solve the structure, with the entire campaign using less than 10 µl protein solution (Harris *et al.*, manuscript in preparation). These examples demonstrate dramatic savings in protein consumption, reagent cost and manpower allocation, allowing concomitant shifts in protein expression and purification methodologies to higher throughput small-scale systems. In this context, a few microlitres of protein, on a par with the amounts often used in sample characterization, become sufficient for a thorough full crystallization screen to scan directly for likelihood to crystallize.

Our study helps to establish the practical application of acoustic liquid transfer to protein crystallization, but also identifies several possible avenues to streamline the workflow towards even smaller volumes. Drop placement is rapid [∼45 s for a single pass over 96 wells at ∼40 nl delivery (see Supplementary Videos S1 and S2^1^)], but mitigation of evaporation will become crucial at smaller volumes. Crystallization trials using 10 nl precipitant solution and 10 nl protein solution were successful, but there was visible evaporation at the perimeter of the precipitant solution prior to the addition of the protein in a second pass. Also, such small volumes highlighted the fact that the more intractable solutions showed higher variability in placement. Optimization of several variables such as enclosure humidification, short droplet flight paths and minimizing the plate volume airspace with new plate geometries should allow the practical use of 5–10 nl protein consumption per experiment or less. This also obviates the need for larger reservoirs. In test cases, we achieved crystal growth even with reservoir volumes of only 1–2 µl (20–40 times larger than the crystallization droplet) set alongside the ∼60 nl crystallization droplet (data not shown); this reduction allows all the experimental liquids including the ‘reservoir’ to be delivered by acoustic liquid transfer.

To crystallographers, the inverted orientation of the target plate immediately suggests the familiar hanging-drop experiment. Given a suitable method for accurately positioning a lid or film seal relative to the ‘source’ plate below, an array of protein droplets could be rapidly set and sealed as hanging drops. The soundwave technology we describe here has the unique capability to eject droplets of the reservoir solutions up to the protein droplet even after the plate has been sealed. A sealed 1536-well plate presents a compelling environment in terms of the issues mentioned above: minimal evaporation, short flight path for accurate drop placement and efficient reagent usage (the acoustic ‘source’ plate becomes the reservoir). Indeed, this provides a very facile non-invasive method for staged addition of precipitant. This real-time modulation could help investigate the trajectory of a droplet on the phase diagram and chart optimal nucleation *versus* post-nucleation crystal-growth regimes (Saridakis & Chayen, 2000 ▶).

The versatile technology is being explored in other creative applications. Rather than transferring microcrystal suspensions to seed crystallization, as we have described elsewhere (Villaseñor *et al.*, 2010 ▶), recent work transferred microcrystals to mesh supports for data collection (Soares *et al.*, 2011 ▶). Furthermore, structure-based drug-discovery or structure-based fragment-screening efforts routinely require significant numbers of small-molecule compounds to be included in cocrystallization or crystal-soaking experiments. With these compounds typically in DMSO a few nanolitres of each can be precisely and accurately added to crystallization drops in under a minute for ∼100 compounds. With the acoustic technology already more common in compound-management groups, shared use of source plates makes efficient use of small-molecule stocks as structural biology groups work through hit lists from high-throughput or fragment-screening campaigns.

We provide a pioneering practical demonstration of the implementation of acoustic liquid transfer in protein crystallization, a particularly challenging application owing to the sheer diversity of the screening space. Our exploration of tuning transfer energies to match liquid properties was here a coarse proof of concept designed to inspire a thorough treatment; more recently both EDC Biosystems and Labcyte have built on this, making progress in more sophisticated acoustic interrogation of each well to establish appropriate energy profiles automatically (Forbush *et al.*, 2006 ▶). These emerging developments obviate the need for users to calibrate and characterize their fluids, providing enhanced accessibility to the technology. Given the excellent reliability of the technology at very small volumes with well characterized liquids such as DMSO (Comley, 2004 ▶), exciting further possibilities and opportunities are poised to be realised as crystallization reagents approach similar performance benchmarks.

## Supplementary Material

Video capture of the inline infrared camera showing drop placement in real time during two stages of a crystal plate setup. Reservoir solution is spotted first, followed by a second pass delivering the protein solution; both set to deliver a nominal 40 nl volume. Owing to a more minimal buffer, the protein component is delivered more rapidly (less variability in focusing the transducer position) and is likely to deliver a slightly larger volume than the more concentrated well solutions at a given fixed energy.. DOI: 10.1107/S0907444912016617/nj5116sup1.mp4

Video capture from the inline infrared camera on the ATS-100 showing drop-on-drop placement with nominal 5 nl droplets.. DOI: 10.1107/S0907444912016617/nj5116sup2.mp4

## Figures and Tables

**Table 1:** Formulations of 33 crystallization solutions (28 unique) that failed to dispense from a total of 480 from the JCSG+, Classics, Index, Wizards and PEG MW screens The ten paired duplicates in this list are marked in bold. 26 of these solutions (21 unique) contained MPD (2-methyl-2,4-pentanediol). JCSG+ No. 79, Classics No. 45 and Wizards No. 88 are marked with an asterisk as these conditions were transferred measurably in another screen. nm, not measured.

Solution No.	Formulation	Viscosity (mPa s)	Density (g cm^−3^)
JCSG+ 41 (137)	**70%(*v*/*v*) MPD, 0.1 *M* HEPES pH 7.5**	21.3	0.98
JCSG+ 11 (107)	**50%(*v*/*v*) MPD, 0.2 *M* ammonium phosphate, 0.1 *M* Tris pH 8.5**	10.2	1.01
JCSG+ 88 (184)	**45%(*v*/*v*) MPD, 0.2 *M* calcium chloride, 0.1 *M* bis-tris pH 5.5**	8.2	1.02
JCSG+ 89 (185)	**45%(*v*/*v*) MPD, 0.2 *M* ammonium acetate, 0.1 *M* bis-tris pH 5.5**	7.7	1
JCSG+ 96 (192)	**45%(*v*/*v*) MPD, 0.2 *M* ammonium acetate, 0.1 *M* HEPES pH 7.5**	7.8	1
JCSG+ 17 (113)	40%(*v*/*v*) MPD, 0.1 *M* cacodylate pH 6.5	14.8	1.01
JCSG+ 44 (140)	40%(*v*/*v*) MPD, 0.1 *M* Tris pH 8	5.6	1
JCSG+ 53 (149)	40%(*v*/*v*) MPD, 0.1 *M* CAPS pH 10.5	6.1	1
JCSG+ 61 (157)	30%(*w*/*v*) Jeffamine M-600, 0.05 *M* cesium chloride, 0.1 *M* MES pH 6.5	5.9	1.03
JCSG+ 37 (133)	24%(*w*/*v*) PEG 1500, 20%(*v*/*v*) glycerol	11.04	1.1
JCSG+ 79 (175)*	15%(*w*/*v*) PEG 3350, 0.1 *M* succinic acid pH 7.0*	4.83	1.03
Classics 22 (22)	**70%(*v*/*v*) MPD, 0.1 *M* HEPES pH 7.5**	19	0.98
Classics 21 (21)	**50%(*v*/*v*) MPD, 0.2 *M* ammonium phosphate, 0.1 *M* Tris pH 8.5**	9.8	1
Classics 17 (17)	30%(*v*/*v*) MPD, 0.2 *M* ammonium acetate, 0.1 *M* sodium citrate pH 5.6	4.4	1.02
Classics 18 (18)	30%(*v*/*v*) MPD, 0.2 *M* magnesium acetate, 0.1 *M* cacodylate pH 6.5	5	1.02
Classics 19 (19)	30%(*v*/*v*) MPD, 0.2 *M* sodium citrate, 0.1 *M* HEPES pH 7.5	5.3	1.04
Classics 20 (20)	30%(*v*/*v*) MPD, 0.5 *M* ammonium sulfate, 0.1 *M* HEPES pH 7.5	4.8	1.04
Classics 45 (45)*	1.6 *M* trisodium citrate*	7.3	1.25
Index 48	**45%(*v*/*v*) MPD, 0.2 *M* calcium chloride dihydrate, 0.1 *M* bis-tris pH 5.5**	nm	nm
Index 49	45%(*v*/*v*) MPD, 0.2 *M* calcium chloride dihydrate, 0.1 *M* bis-tris pH 6.5	nm	nm
Index 50	**45%(*v*/*v*) MPD, 0.2 *M* ammonium acetate, 0.1 *M* bis-tris pH 5.5**	nm	nm
Index 51	45%(*v*/*v*) MPD, 0.2 *M* ammonium acetate, 0.1 *M* bis-tris pH 6.5	nm	nm
Index 52	**45%(*v*/*v*) MPD, 0.2 *M* ammonium acetate, 0.1 *M* HEPES pH 7.5**	nm	nm
Index 53	45%(*v*/*v*) MPD, 0.2 *M* ammonium acetate, 0.1 *M* Tris pH 8.5	nm	nm
Index 58	45%(*v*/*v*) polypropylene glycol P 400, 0.1 *M* bis-tris pH 6.5	nm	nm
Index 56	35%(*v*/*v*) pentaerythritol propoxylate, 0.2 *M* potassium chloride, 0.05 *M* HEPES pH 7.5	nm	nm
Wizards 4	35%(*v*/*v*) MPD, 0.2 *M* magnesium chloride, 0.1 *M* imidazole pH 8.0	nm	nm
Wizards 24	35%(*v*/*v*) MPD, 0.2 *M* sodium chloride, 0.1 *M* Tris pH 7.0	nm	nm
Wizards 43	35%(*v*/*v*) MPD, 0.1 *M* sodium/potassium phosphate pH 6.2	nm	nm
Wizards 50	35%(*v*/*v*) MPD, 0.2 *M* lithium sulfate, 0.1 *M* MES pH 6.0	nm	nm
Wizards 69	35%(*v*/*v*) MPD, 0.1 *M* sodium acetate pH 4.5	nm	nm
Wizards 73	35%(*v*/*v*) MPD, 0.2 *M* sodium chloride, 0.1 *M* HEPES pH 7.5	nm	nm
Wizards 88*	20%(*w*/*v*) PEG 3000, 0.2 *M* zinc acetate, 0.1 *M* imidazole pH 8.0*	nm	nm

**Table 2:** Aggregate and ‘by screen’ statistics of droplet volume distribution Mean volume (nl) and standard deviation (SD) are shown to indicate bias and variance (conditions that did not dispense were excluded). Root-mean-square deviations (r.m.s.d.s) of the residuals (relative to 5 nl target volume) are calculated as a gauge of overall accuracy.

Screen	Energy profile	Mean volume ± SD (r.m.s.d.)
JCSG+	Fixed	4.81 ± 1.17 (1.18)
	Tuned	5.41 ± 0.52 (0.66)
Classics	Fixed	5.46 ± 1.69 (1.74)
	Tuned	5.59 ± 0.91 (1.08)
Wizards	Fixed	5.14 ± 1.22 (1.22)
	Tuned	5.94 ± 0.85 (1.26)
PEG MW	Fixed	5.46 ± 0.82 (0.94)
	Tuned	5.75 ± 0.59 (0.95)
Aggregate	Fixed	5.23 ± 1.28 (1.30)
	Tuned	5.67 ± 0.76 (1.01)

## Footnotes

1 Supplementary material has been deposited in the IUCr electronic archive (Reference: NJ5116). Services for accessing this material are described at the back of the journal.

## References

[bb1] Aerni, H. R., Cornett, D. S. & Caprioli, R. M. (2006). *Anal. Chem.* **78**, 827–834. 10.1021/ac051534r 16448057

[bb2] Bodenstaff, E. R., Hoedemaeker, F. J., Kuil, M. E., de Vrind, H. P. M. & Abrahams, J. P. (2002). *Acta Cryst.* D**58**, 1901–1906. 10.1107/s0907444902016608 12393920

[bb3] Comley, J. (2004). *Drug Discov. World*, **Summer 2004**, 43–54.

[bb17] Ellson, R., Stearns, R., Mutz, M., Brown, C., Browning, B., Harris, D., Qureshi, S., Shieh, J. & Wold, D. (2005). *Comb. Chem. High Throughput Screen.* **8**, 489–498. 10.2174/1386207054867382 16178808

[bb4] Forbush, M., Chow, H., Chiao, J. & Rose, A. (2006). *J. Assoc. Lab. Autom* **11**, 188–194.

[bb5] Kim, J. L., Morgenstern, K. A., Griffith, J. P., Dwyer, M. D., Thomson, J. A., Murcko, M. A., Lin, C. & Caron, P. R. (1998). *Structure*, **6**, 89–100. 10.1016/s0969-2126(98)00010-0 9493270

[bb6] Kutach, A. K., Villaseñor, A. G., Lam, D., Belunis, C., Janson, C., Lok, S., Hong, L.-N., Liu, C.-M., Deval, J., Novak, T. J., Barnett, J. W., Chu, W., Shaw, D. & Kuglstatter, A. (2010). *Chem. Biol. Drug Des.* **76**, 154–163. 10.1111/j.1747-0285.2010.00993.x 20545945

[bb7] Le Pogam, S. *et al.* (2006). *J. Virol.* **80**, 6146–6154. 10.1128/JVI.02628-05 PMC1472602 16731953

[bb8] Newman, J., Fazio, V. J., Lawson, B. & Peat, T. S. (2010). *Cryst. Growth Des.* **10**, 2785–2792.

[bb18] Olechno, J., Shieh, J. & Ellson, R. (2006). *J. Assoc. Lab. Autom.* **11**, 240–246.

[bb9] Rose, D. (1999). *Drug Discov. Today*, **4**, 411–419. 10.1016/s1359-6446(99)01388-4 10461151

[bb10] Saridakis, E. & Chayen, N. E. (2000). *Protein Sci.* **9**, 755–757. 10.1110/ps.9.4.755 PMC2144611 10794418

[bb11] Soares, A. S., Engel, M. A., Stearns, R., Datwani, S., Olechno, J., Ellson, R., Skinner, J. M., Allaire, M. & Orville, A. M. (2011). *Biochemistry*, **50**, 4399–4401. 10.1021/bi200549x PMC3144476 21542590

[bb12] Stevens, R. C. (2000). *Curr. Opin. Struct. Biol.* **10**, 558–563. 10.1016/s0959-440x(00)00131-7 11042454

[bb13] Sweeney, Z. K. *et al.* (2008). *Bioorg. Med. Chem. Lett.* **18**, 4352–4354. 10.1016/j.bmcl.2008.06.072 18632268

[bb14] Villaseñor, A. G., Wong, A., Shao, A., Garg, A., Kuglstatter, A. & Harris, S. F. (2010). *Acta Cryst.* D**66**, 568–576. 10.1107/S0907444910005512 20445232

[bb15] Walter, T. S., Diprose, J., Brown, J., Pickford, M., Owens, R. J., Stuart, D. I. & Harlos, K. (2003). *J. Appl. Cryst.* **36**, 308–314.

[bb16] Wong, E. Y. & Diamond, S. L. (2009). *Anal. Chem.* **81**, 509–514. 10.1021/ac801959a PMC3220162 19035650

